# New York Polyclinic Medical School and Hospital

**Published:** 1912-04

**Authors:** 


### New York Polyclinic Medical School and Hospital.

Meeting of February 5, 1912.

A Case of Advanced Carcinosis: Prolongation of Life by Operation.—Presented by Dr. C. A. Frink.—Dr. Frink presented a case of a woman, 48 years of age, widow with three children, one miscarriage. Her family history showed longevity on both sides. Her husband died of phthisis twenty-six years ago. She had one previous attack of appendicitis. Seventeen months ago patient had attacks of what she called indigestion, with constant pain behind the sternum, and vomiting. Never vomited blood, but noticed that food taken several days previously appeared in the vomitus. A diagnosis by her physician was made of "nervous dyspepsia." She lost weight and strength and the vomiting increased. No lung symptoms were present. On entering the Polyclinic Hospital she could not retain food, and she showed a stenosis of the pyloric valve. She was very emaciated, skin dry, and no adipose tissue at all. Laboratory findings: Blood, a secondary anæmia; sputum, negative. The X-Ray was unsatisfactory. Stomach contents showed presence of lactic acid, no free HCl or Boas bacilli. Urine, normal.

Examination of the abdomen showed a mass the size of an orange, situated over the pyloric valve, immovable. Operation, December 4, 1908, by Dr. Bainbridge, who did a retro-colic gastro-jejunostomy. The inoperable mass with its enlarged glands which about closed off the pyloris were not touched.

Subsequent History: June 11, 1909. The patient returned with her first trouble since operation. Vomiting after food. She otherwise continued in good health until the spring of 1911. In May of this year she showed signs of infection by tuberculosis. And in July the tubercular bacilli were found in the sputum. The patient died of pulmonary tuberculosis on August 6, 1911.

Conclusion: The history of this case emphasizes the importance of operating on cases of cancer that do not appear from the clinical findings to be good surgical risks. This patient was a poor surgical risk so far as there being any chance of curing her condition by operation. Nevertheless by doing all in our power, she was able to return to her home and family, enjoying good health, eating and sleeping well, and she gained over fifteen pounds in weight. Two and one-half years after operation she died of a condition entirely independent of her former trouble. We feel that through surgical intervention we prolonged this woman's life, making her a useful member of society for this additional time.

Dr. John A. Wyeth said he considered it the duty of a surgeon to take any and all risks regardless of what might be the result on his reputation or statistics when the patient is in such a state that the conditions seem absolutely hopeless and death seems imminent, no matter whether the patient dies on the table or not, if in the judgment of the conscientious surgeon there is a possibility of contributing to the patient's comfort, lessening his discomfort or prolonging his life, it is his duty to undertake the operation. One of the severest criticisms I could make about any man is that he would not undertake a case if he thought the patient would die. Dr. Bainbridge said that at the time of operating on this case he had met the conditions exactly as Dr. Wyeth had described. The patient had consented knowing well her serious condition, and had never ceased to be grateful for her prolonged life. She seemed particularly discouraged in not being able to survive the phthisis when she had been relieved of the stomach condition which seemed to her to be so much worse.

A Case of Tubercular Peritonitis: Prolongation of Life by Intra-Abdominal Administration of Oxygen.—By Dr. H. D. Meeker.—Dr. Meeker showed a case of tubercular peritonitis which he had treated by the intra-abdominal administration of oxygen. The case was apparently cured. He also advocated its use in cases of profound shock and ascitis, and said that it required from seventy-two hours to four or five days for complete absorption. Care had to be exercised in watching cases as the abdomen became flat in from forty-eight to seventy-two hours. Collapse from its complete absorption should be guarded against by the free administration of stimulants. Dr. Bainbridge said he had been using oxygen to meet shock in abdominal surgery for the past eight years, and had treated in all about one hundred cases. He had noted marked improvement and a ready response to its use.

A New Pneumatic Electric Proctoscope.—Shown and Demonstrated by Dr. Frank C. Yeomans.—Dr. Yeomans demonstrated a new proctoscope and sigmoidoscope. He said that hitherto the practical methods of directing lighting of protoscopes were by small electric bulbs, carried near the distal ends of the tubes on insulated carriers, and that they burned out easily. He had adopted the principle of illumination by a more powerful light within and at the end of the ocular portion of the tube. The proctoscope is ten inches long, graduated in inches seven-eighths of an inch in diameter, and is fitted with a large flange at the proximal end, which is perforated by a small tube, joining the main tube at an angle. A light carrier fits very tightly into the auxiliary tube, and a substantial incandescent bulb is covered with a capsule bearing a plano-convex lense so set the collected rays are refracted at a compensating angle to the light carrier. This lense only projects into the main tube, and in no practical way interferes with inspection of the bowel or the passing of instruments.

The ocular end of the tube is enclosed hermetically by a plug which contains a glass window to magnify the illuminated field. A hand bulb attached to a small offset at the side of the plug inflates the bowel to any degree desired. The same light carrier and plug fit the sigmoidoscope, which is also fourteen inches long and three-fourths of an inch in diameter.

The points of superiority in this new instrument are: Simplicity of construction; sterilizable by boiling; excellent illumination by a strong electric lamp which will not burn out readily; and thorough practicability for examination, diagnosis or treatment of lesions in the rectum or sigmoid below its apex.

